# Individualized prediction of survival benefit from primary tumor resection for patients with unresectable metastatic colorectal cancer

**DOI:** 10.1186/s12957-020-01972-y

**Published:** 2020-08-03

**Authors:** Yi Yang, Yujie Lu, Wen Jiang, Jinzhou Zhu, Su Yan

**Affiliations:** 1 Department of Gastroenterology, The First Affiliated Hospital of Soochow University, 899 Pinghai Road, Suzhou, 215006 Jiangsu China; 2 Department of Oncology, The First Affiliated Hospital of Soochow University, 899 Pinghai Road, Suzhou, 215006 Jiangsu China

**Keywords:** Nomogram, Survival, SEER, Colorectal cancer, Metastasis, Primary tumor resection

## Abstract

**Background:**

The impact of primary tumor resection (PTR) on the prognosis of unresectable metastatic colorectal cancer (mCRC) patients remains debatable. We aimed to develop several prognostic nomograms which could be useful in predicting whether patients might benefit from PTR or not.

**Methods:**

Patients diagnosed as mCRC without resected metastasis were identified from the Surveillance Epidemiology and End Results database and randomly assigned into two groups: a training cohort (6369 patients) and a validation cohort (2774 patients). Univariate and multivariable Cox analyses were performed to identify the independent predictors and construct nomograms that could independently predict the overall survival (OS) of unresectable mCRC patients in PTR and non-PTR groups, respectively. The performance of these nomograms was assessed by the concordance index (C-index), calibration curves, and decision curve analysis (DCA).

**Results:**

Based on the result of univariate and multivariable Cox analyses, two nomograms were respectively constructed to predict the 1-year OS rates of unresectable mCRC patients when receiving PTR and not. The first one included age, gender, tumor grade, proximal colon, N stage, CEA, chemotherapy, radiotherapy, histology type, brain metastasis, liver metastasis, lung metastasis, and bone metastasis. The second nomogram included age, race, tumor grade, primary site, CEA, chemotherapy, brain metastasis, and bone metastasis. These nomograms showed favorable sensitivity with the C-index range of 0.700–0.725. The calibration curves and DCAs also exhibited adequate fit and ideal net benefits in prognosis prediction and clinical application.

**Conclusions:**

These practical prognosis nomograms could assist clinicians in making appropriate treatment decisions to effectively manage the disease.

## Introduction

Colorectal cancer (CRC) is one of the most frequently diagnosed malignancies and is ranked second common cause of cancer-related death worldwide [1]. Although remarkable progress has been made in the development of chemotherapy and molecular targeting drugs in recent years [2], surgical resection remains the prioritize option for non-metastatic CRC [3]. Nevertheless, approximately 22% of CRC patients have diagnosed with synchronous distant metastasis [4], and more than 70% of distant metastatic disease could not be radically resected [5].

Given that most mCRC are currently incurable, treatment is meant to help patients achieve a high quality of life and increased life expectancy. According to the American Society of Colon and Rectal Surgeons (ASCRS) and National Comprehensive Cancer Network (NCCN) guidelines, the standard treatment for unresectable mCRC is systemic chemotherapy, and primary tumor resection (PTR) is only recommended for patients with fatal complications, such as bleeding, perforation, or obstruction [6, 7]. Although chemotherapy regimens and new targeted agents may be effective controlling the primary lesions for most mCRC patients, disease progression has been reported in many patients after several months or 1–2 years. Previous studies suggest that PTR can effectively prevent and reduce the serious tumor-related complications, thereby reducing the risk of death [8–12]. However, some factors which seriously affect survival, such as the delay of chemotherapy and postoperative complications, should also be taken into consideration [13–16]. So far, mixed conclusions have been reported regarding the PTR procedure [5, 8–12, 17–22]. Besides, clinicians are still ambivalent about the effect of PTR in unresectable mCRC patients. Therefore, a practical and customized approach is needed to enable clinicians to make accurate treatment decisions by considering the potential risks and benefits of PTR.

Therefore, this study used a large population-based data to determine independent prognostic predictors of unresectable mCRC patients who underwent PTR and who did not, respectively. Several individualized nomograms were further developed based on these factors to respectively predict the survival of unresectable mCRC patients with or without PTR. It is expected that those practical prognostic nomograms may assist in personalized predictions of the survival of patients when receiving and not receiving PTR before surgery, thereby indicating whether the patients may benefit from the PTR.

## Materials and methods

### Database and patient selection

**Fig. 1:**
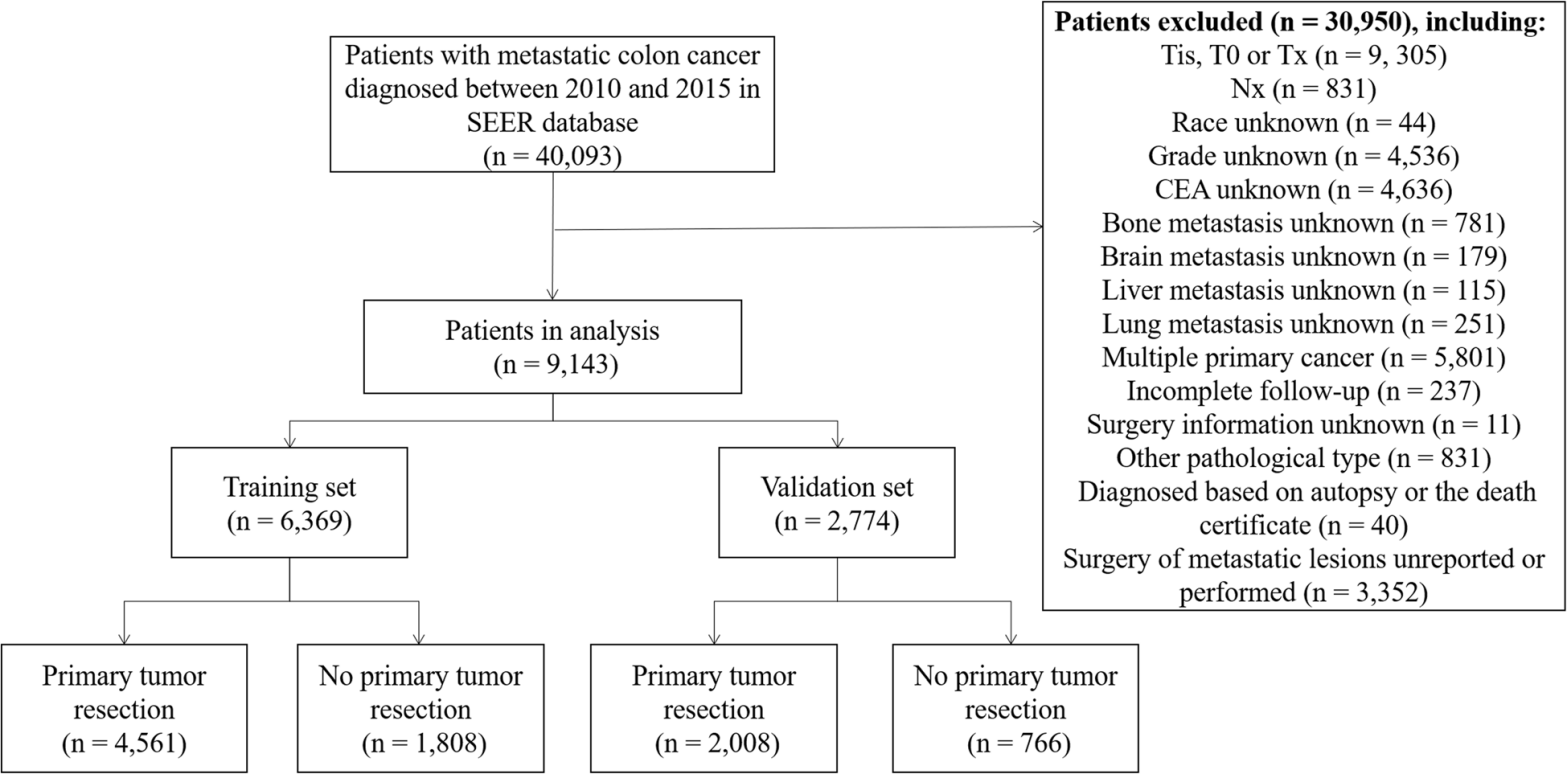
Flowchart of data selection from Surveillance, Epidemiology, and End Results (SEER) database

We retrieved data from the American National Cancer Institute Surveillance, Epidemiology, and End Results (SEER) database, which covers more than 28% of the American population. Patients with mCRC cancer who were pathologically confirmed diagnosed from 2010 to 2015 were identified in the present study. The International Classification of Diseases for Oncology 3rd edition (ICD-O-3) was used to limit the pathology types to adenocarcinoma (8140-8147, 8210-8211, 8220-8221, and 8260-8263), mucinous adenocarcinoma (MAC) (8480-8481), and signet ring cell carcinoma (SRCC) (8490). Exclusion criteria consisted of the following: (1) stage Tis, T0, Tx, or NX; (2) unknown histological grade; (3) unknown race; (4) unknown CEA status; (5) unknown information of distant metastasis; (6) incomplete follow-up; (7) multiple primary cancer; (8)unknown surgery information; (9) diagnosis based on autopsy or the death certificate; (10) other pathological types; and (11) surgery of metastatic sites unreported or performed. Finally, we recruited 9143 mCRC patients in this study, who were divided randomly into two cohorts (7:3): the training cohort (6369 patients), and the validation cohort (2774 patients). Each cohort was further divided into two groups according to the PTR status. The patient selection process was shown in Fig. 1. Given that the SEER database is an open-access cancer database that only contains de-identified patient data, this study was exempted from the approval of the institutional review board of the First Affiliated Hospital of Soochow University.

### Variables and outcomes

Data on demographic characteristics of patients (age at diagnosis, gender, race, and year of diagnosis), tumor and treatment characteristics (tumor grade, histology types, primary tumor sites, distant metastatic sites, T and N stage, CEA, radiotherapy, chemotherapy, and surgery), and survival data (survival time, survival status, and cause of death) were analyzed. Patients’ follow-up ended in December 2016 or upon death. Age, as a continuous variable, was divided into two categories (< 60 and ≥ 60). We classified primary tumor sites as proximal colon (C18.0, C18.2-18.5), distal colon (C18.6-18.7), rectum (C19.0-C19.9), and other sites (C18.8-18.9). The outcomes were overall survival (OS) and cancer-specific survival (CSS), which were respectively defined as the time from diagnosis to death for any reason and the time from diagnosis to death for mCRC.

### Statistical analysis

Chi-square test was used to compare the difference of baseline clinical characteristics between the training and validation cohorts, as well as PTR and non-PTR groups. Kaplan–Meier method and the log-rank test were used to compare the statistical difference of OS and CSS between PTR and non-PTR groups. Multivariable Cox proportional hazard regression analyses containing the covariates, including age at diagnosis, gender, race, T and N stage, tumor grade, histology types, primary tumor sites, distant metastatic sites (liver, bone, brain, and lung), CEA level and treatment characteristics (radiotherapy and chemotherapy), were used to assess the survival benefits of PTR. Further, univariate and multivariable Cox regression analyses were used to identify independent prognostic factors based on the training cohort. Variables in the nomograms were selected based on the multivariable Cox regression analyses and the minimum Akaike Information Criterion (AIC) values.

Several nomograms were developed to predict 1-year OS and CSS in PTR and non-PTR groups, respectively. The predictive performances of these nomograms, including predictive accuracy and calibration, were evaluated both in the training and validation cohorts. The concordance index (C-index) was employed to measure the predictive accuracy. The calibration was assessed graphically by calibration curves, which represented the agreement between observed outcome and predicted probabilities. We also used the decision curve analysis (DCA) to evaluate the clinical usefulness in all patients, thereby quantifying the net benefits at different threshold probabilities.

The data was extracted using the SEER*Stat software (version 8.3.5; http://seer.cancer.gov/seerstat/). All statistical analyses were performed using the R software (version 3.5.2; http://www.r-project.org) and the SPSS statistics software (version 21; IBM Corp, Armonk, NY). The two-tailed *p* value < 0.05 was set as the statistical significance level for all statistics.

## Result

### Patient clinicopathological characteristics

After the strict selecting, a total of 9143 unresectable mCRC patients without radical resection from 2010 to 2015 in the SEER database were finally included. The median age was 62 years (interquartile range, 53–72), and gender distribution was 5145 males (56.27%) and 3998 females (43.73%). For the sites of metastasis, 73.73%, 0.93%, 23.80%, and 4.41% of the patients had liver metastasis, brain metastasis, lung metastasis, and bone metastasis, respectively. Through random assignment, 6369 patients were enrolled in the training cohort and 2774 in the validation cohort. A significant difference was observed only in the distribution of radiotherapy between the two cohorts (*p* = 0.006). The clinical data of these two cohorts are shown in Table 1.

**Table 1:** Difference of patient characteristics in training and validation cohorts

Variables	Total population	Training cohort	Validation cohort	*p* value
	(*n* = 9143)	(*n* = 6369)	(*n* = 2774)	
Age *n* (%)				
< 60	3891 (42.56%)	2700 (42.39%)	1191 (42.93%)	0.630
≥ 60	5252 (57.44%)	3669 (57.61%)	1583 (57.07%)	
Gender *n* (%)				
Male	5145 (56.27%)	3595 (56.45%)	1550 (55.88%)	0.614
Female	3998 (43.73%)	2774 (43.55%)	1224 (44.12%)	
Race *n* (%)				
Black	6838 (74.79%)	4771 (74.91%)	2067 (74.51%)	0.919
White	1441 (15.76%)	1000 (15.70%)	441 (15.90%)	
Other	864 (9.45%)	598 (9.39%)	266 (9.59%)	
Grade *n* (%)				
I and II	6614 (72.34%)	4613 (72.43%)	2001 (72.13%)	0.772
III and IV	2529 (27.66%)	1756 (27.57%)	773 (27.87%)	
Primary site *n* (%)				
Proximal colon	3830 (41.89%)	2653 (41.65%)	1177 (42.43%)	0.630
Distal colon	2441 (26.70%)	1690 (26.53%)	751 (27.07%)	
Rectum	2694(29.47%)	1903 (29.88%)	791(28.51%)	
Other	178 (1.95%)	123 (1.93%)	55 (1.98%)	
Histology type *n* (%)				
AC	8314 (90.93%)	5784 (90.81%)	2530 (91.20%)	0.379
MAC	655 (7.16%)	469 (7.36%)	186 (6.71%)	
SRCC	174 (1.90%)	116 (1.82%)	58 (2.09%)	
CEA *n* (%)				
Normal	1763 (19.28%)	1227 (19.27%)	536 (19.32%)	0.949
Abnormal	7380 (80.72%)	5142 (80.73%)	2238 (80.68%)	
Radiotherapy *n (%)*				
No	7930 (86.73%)	5483 (86.09%)	2447 (88.21%)	0.006
Yes	1213 (13.27%)	886 (13.91%)	327 (11.79%)	
Chemotherapy *n* (%)				
No	2461 (26.92%)	1681 (26.39%)	780 (28.12%)	0.087
Yes	6682 (73.08%)	4688 (73.61%)	1994 (71.89%)	
Surgery type *n* (%)				
Non-PTR	2574 (28.15%)	1808 (28.39%)	766 (27.61%)	0.449
PTR	6569 (71.85%)	4561 (71.61%)	2008 (72.39%)	
T stage *n* (%)				
T1 and T2	1374 (15.03%)	969 (15.21%)	405 (14.60%)	0.450
T3 and T4	7769 (84.97%)	5400 (84.79%)	2369 (85.40%)	
N stage *n* (%)				
N0	2398 (26.23%)	1685 (26.46%)	713 (25.70%)	0.753
N1	3511 (38.40%)	2439 (38.29%)	1072 (38.64%)	
N2	3234 (35.37%)	2245 (35.25%)	989 (35.65%)	
Bone metastasis *n* (%)				
No	8740 (95.59%)	6087 (95.57%)	2653 (95.64%)	0.888
Yes	403 (4.41%)	282 (4.43%)	121 (4.36%)	
Liver metastasis *n* (%)				
No	2402 (26.27%)	1670 (26.22%)	732 (26.39%)	0.867
Yes	6741 (73.73%)	4699 (73.78%)	2042 (73.61%)	
Lung metastasis *n* (%)				
No	6967 (76.20%)	4850 (76.15%)	2117 (76.32%)	0.864
Yes	2176 (23.80%)	1519 (23.85%)	657 (23.68%)	
Brain metastasis *n* (%)				
No	9058 (99.07%)	6309 (99.06%)	2749 (99.10%)	0.852
Yes	85 (0.93%)	60 (0.94%)	25 (0.90%)	

*AC* adenocarcinoma, *MAC* mucinous adenocarcinoma, *SRCC* signet ring cell carcinoma, *PTR* primary tumor resection

Among the 6369 unresectable mCRC patients in the training cohort, 4561 patients had received PTR, while 1808 patients did not receive any cancer-directed surgery (Table 2). Race did not differ between the PTR group and non-PTR group (*p* = 0.859); however, there were significant differences between the two groups for age, gender, tumor grade, primary site, histology type, lung metastasis, liver metastasis, brain metastasis, and bone metastasis (all *p* < 0.05). The patients who received PTR were more likely to be higher T stage, higher N stage, and the normal level of CEA (all *p* < 0.05) compared to patients who did not. The significant differences were also observed in the chemotherapy and radiotherapy between the PTR group and non-PTR group (all *p* < 0.05).

**Table 2:** Demographics and disease characteristics of patients in the training cohort

Variables	PTR	Non-PTR	*p* value
	(*n* = 4561)	(*n* = 1808)	
Age *n* (%)			
< 60	1881 (41.24%)	819 (45.30%)	0.003
≥ 60	2680 (58.76%)	989 (54.70%)	
Gender *n* (%)			
Male	2496 (54.72%)	1099 (60.79%)	< 0.001
Female	2065 (45.28%)	709 (39.21%)	
Race *n* (%)			
Black	3424 (75.07%)	1347 (74.50%)	0.859
White	714 (15.65%)	286 (15.82%)	
Other	423 (9.27%)	175 (9.68%)	
Grade *n* (%)			
I and II	3226 (70.73%)	1387 (76.71%)	< 0.001
III and IV	1335 (29.27%)	421 (23.29%)	
Primary site *n* (%)			
Proximal colon	2210 (8.45%)	443 (24.50%)	< 0.001
Distal colon	1335 (29.27%)	355 (19.63%)	
Rectum	921 (20.19%)	982 (54.31%)	
Other	95 (2.08%)	28 (1.55%)	
Histology type *n* (%)			
AC	4071 (89.26%)	1713 (94.75%)	< 0.001
MAC	412 (9.03%)	57 (3.15%)	
SRCC	78 (1.71%)	38 (2.10%)	
CEA *n* (%)			
Normal	990 (21.71%)	237 (13.11%)	< 0.001
Abnormal	3571 (78.29%)	1571 (86.89%)	
Radiotherapy *n* (%)			
No	4103 (89.96%)	1380 (76.33%)	< 0.001
Yes	458 (10.04%)	428 (23.67%)	
Chemotherapy *n* (%)			
No	1272 (27.89%)	409 (22.62%)	< 0.001
Yes	3289 (72.11%)	1399 (77.38%)	
T stage *n* (%)			
T1 and T2	207 (4.54%)	762 (42.15%)	< 0.001
T3 and T4	4354 (95.46%)	1046 (57.85%)	
N stage *n* (%)			
N0	796 (17.45%)	889 (49.17%)	< 0.001
N1	1656 (36.31%)	783 (43.31%)	
N2	2109 (46.24%)	136 (7.52%)	
Bone metastasis *n* (%)			
No	4421 (96.93%)	1666 (92.15%)	< 0.001
Yes	140 (3.07%)	142 (7.85%)	
Liver metastasis *n* (%)			
No	1254 (27.49%)	416 (23.01%)	< 0.001
Yes	3307 (72.51%)	1392 (76.99%)	
Lung metastasis *n* (%)			
No	3683 (80.75%)	1167 (64.55%)	< 0.001
Yes	878 (19.25%)	641 (35.45%)	
Brain metastasis *n* (%)			
No	4531 (99.34%)	1778 (98.34%)	< 0.001
Yes	30 (0.66%)	30 (1.66%)	

*AC* adenocarcinoma, *MAC* mucinous adenocarcinoma, *SRCC* signet ring cell carcinoma, *PTR* primary tumor resection

### Analysis of survival benefits from surgery

**Fig. 2:**
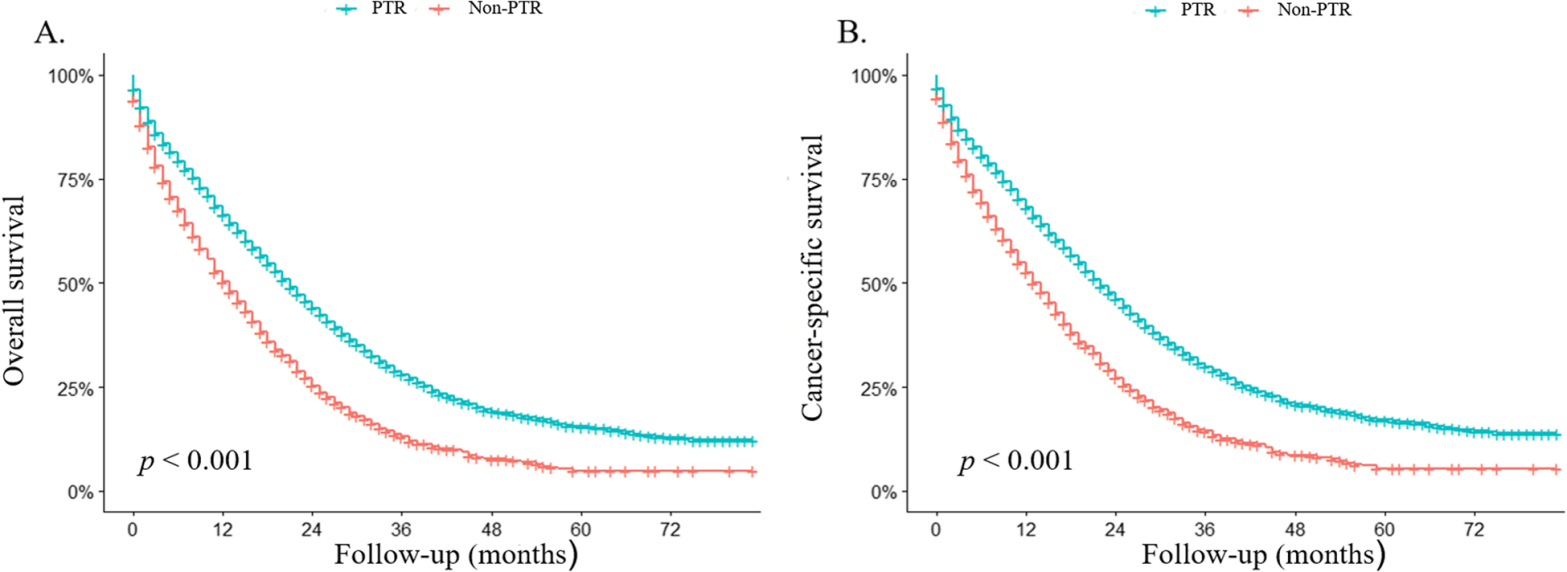
Effect of primary tumor resection on **a** overall survival and **b** cancer-specific survival in unresectable metastatic colorectal cancer

At the time of the last follow-up, 4762 patients had died, with CRC being the cause of death in 4505 cases. The Kaplan–Meier curves demonstrated that patients with PTR had a better OS (Fig. 2a, *p* < 0.001) and CSS (Fig. 2b, *p* < 0.001) compared with the non-PTR group. After adjusting for age at diagnosis, gender, race, T and N stage, tumor grade, histology types, primary tumor sites, distant metastatic sites (liver, bone, brain, and lung), CEA level, and treatment characteristics (radiotherapy and chemotherapy), PTR was associated with approximately 54.2% and 54.8% relative reduction in overall mortality (HR = 0.458, 95% CI 0.422–0.497; *p* < 0.001) and cancer-specific mortality (HR = 0.452, 95% CI 0.415–0.492; *p* < 0.001), respectively (Table 3). To further confirm the impact of PTR on the survival in different specific subgroups, multivariable Cox analyses by subgroups were conducted (Table 3). The results of the subgroup analyses demonstrated that PTR exerted significantly improved OS and CCS in almost all subgroups except for OS in the brain metastasis group (*p* = 0.065).

**Table 3:** Subgroup multivariable Cox analyses of the impact of primary tumor resection on overall survival and cancer-specific survival

Variables	OS	*p* value^*^	CSS	*p* value^*^
	HR (95% CI)		HR (95% CI)	
Total	0.458 (0.422–0.497)	< 0.001	0.452 (0.415–0.492)	< 0.001
Age				
< 60	0.477 (0.421–0.542)	< 0.001	0.477 (0.419–0.542)	< 0.001
≥ 60	0.449 (0.402–0.500)	< 0.001	0.438 (0.392–0.491)	< 0.001
Gender				
Male	0.453 (0.408–0.504)	< 0.001	0.444 (0.398–0.495)	< 0.001
Female	0.464 (0.407–0.529)	< 0.001	0.463 (0.404–0.530)	< 0.001
Race				
Black	0.459 (0.417–0.505)	< 0.001	0.453 (0.411–0.499)	< 0.001
White	0.419 (0.340–0.516)	< 0.001	0.424 (0.341–0.526)	< 0.001
Other	0.510 (0.385–0.675)	< 0.001	0.476 (0.356–0.635)	< 0.001
Grade				
I and II	0.449 (0.408–0.494)	< 0.001	0.443 (0.401–0.489)	< 0.001
III and IV	0.477 (0.404–0.562)	< 0.001	0.473 (0.399–0.561)	< 0.001
Primary site				
Proximal colon	0.395 (0.341–0.458)	< 0.001	0.389 (0.335–0.453)	< 0.001
Distal colon	0.359 (0.302–0.426)	< 0.001	0.345 (0.289–0.412)	< 0.001
Rectum	0.563 (0.499–0.637)	< 0.001	0.558 (0.492–0.634)	< 0.001
Other	0.341 (0.186–0.627)	0.001	0.358 (0.193–0.666)	0.001
Histology type				
AC	0.455 (0.418–0.496)	< 0.001	0.448 (0.410–0.489)	< 0.001
MAC	0.386 (0.262–0.569)	< 0.001	0.396 (0.265–0.591)	< 0.001
SRCC	0.396 (0.204–0.769)	0.006	0.384 (0.195–0.758)	0.006
CEA				
Normal	0.336 (0.272–0.415)	< 0.001	0.320 (0.258–0.398)	< 0.001
Abnormal	0.484 (0.442–0.529)	< 0.001	0.481 (0.439–0.528)	< 0.001
Radiotherapy				
No	0.464 (0.423–0.509)	< 0.001	0.453 (0.412–0.498)	< 0.001
Yes	0.420 (0.348–0.507)	< 0.001	0.435 (0.358–0.529)	< 0.001
Chemotherapy				
No	0.471 (0.401–0.554)	< 0.001	0.454 (0.384–0.537)	< 0.001
Yes	0.469 (0.427–0.516)	< 0.001	0.466 (0.422–0.514)	< 0.001
T stage				
T1 and T2	0.436 (0.351–0.541)	< 0.001	0.429 (0.343–0.536)	< 0.001
T3 and T4	0.463 (0.422–0.507)	< 0.001	0.457 (0.416–0.502)	< 0.001
N stage				
N0	0.394 (0.342–0.455)	< 0.001	0.383 (0.330–0.444)	< 0.001
N1	0.451 (0.399–0.510)	< 0.001	0.445 (0.393–0.505)	< 0.001
N2	0.649 (0.519–0.812)	< 0.001	0.663 (0.527–0.836)	< 0.001
Bone metastasis				
No	0.455 (0.418–0.496)	< 0.001	0.448 (0.411–0.489)	< 0.001
Yes	0.426 (0.295–0.616)	< 0.001	0.453 (0.310–0.662)	< 0.001
Liver metastasis				
No	0.371 (0.312–0.440)	< 0.001	0.358 (0.299–0.428)	< 0.001
Yes	0.493 (0.449–0.542)	< 0.001	0.488 (0.443–0.538)	< 0.001
Lung metastasis				
No	0.423 (0.384–0.466)	< 0.001	0.420 (0.380–0.464)	< 0.001
Yes	0.567 (0.485–0.664)	< 0.001	0.550 (0.468–0.646)	< 0.001
Brain metastasis				
No	0.457 (0.421–0.497)	< 0.001	0.452 (0.415–0.492)	< 0.001
Yes	0.400 (0.151–1.060)	0.065	0.338 (0.122–0.932)	0.036

All HRs refer to surgery versus non-surgery (reference) in the subgroup analysis

*CI* confidence interval, *AC* adenocarcinoma, *MAC* mucinous adenocarcinoma, *SRCC* signet ring cell carcinoma

*Multivariant Cox regression model

### Risk factors related to survival in cohorts with and without PTR

Univariate Cox regression analyses revealed that age at diagnosis, tumor grade, primary site, CEA, radiotherapy, chemotherapy, N stage, histology type, bone metastasis, and brain metastasis were correlated with OS and CCS in both PTR group and non-PTR group (all *p* < 0.05, Table 4). In addition, race was only a risk factor for poorer OS and CSS in the non-PTR group, while gender, T stage, liver metastasis, and lung metastasis were only the significant prognostic factors (OS and CCS) in the PTR group (all *p* < 0.05, Table 4).These significant factors associated with OS and CSS identified in univariate Cox regression analyses were further subjected to multivariable analyses. In the non-PTR group, multivariable Cox regression analyses revealed eight variables (age, race, tumor grade, primary site, CEA, chemotherapy, brain metastasis, and bone metastasis) were significantly associated with OS of patients, and seven variables (age, race, tumor grade, primary site, CEA, chemotherapy, and bone metastasis) were significantly associated with CCS (all *p* < 0.05, Table 5). In PTR group, 13 variables (older age, female, poorer tumor grade, proximal colon, higher N stage, the abnormal value of CEA, no chemotherapy, no radiotherapy, MAC and SRCC, brain metastasis, liver metastasis, lung metastasis, and bone metastasis) were identified as the independent risk factors associated with OS and CCS (all *p* < 0.05, Table 5).

**Table 4:** Univariate Cox regression analyses for metastasis colorectal cancer patients in PTR and non-PTR groups

Variables	Overall survival							Cancer-specific survival						
	Non-PTR				PTR			Non-PTR				PTR		
	HR (95% CI)		*p* value		HR (95% CI)		*p* value	HR (95% CI)		*p* value		HR (95% CI)		*p* value
Age														
< 60	Reference				Reference			Reference				Reference		
≥ 60	1.519 (1.370–1.684)		< 0.001		1.529 (1.423–1.641)		< 0.001	1.502 (1.350–1.670)		< 0.001		1.495 (1.390–1.608)		< 0.001
Gender														
Male	Reference				Reference			Reference				Reference		
Female	1.033 (0.931–1.147)		0.538		1.108 (1.034–1.187)		0.003	1.037 (0.931–1.154)		0.510		1.111 (1.035–1.192)		0.004
Race														
Black	Reference				Reference			Reference				Reference		
White	1.237 (1.078–1.420)		0.002		1.027 (0.934–1.129)		0.583	1.223 (1.061–1.410)		0.005		1.035 (0.939–1.140)		0.490
Other	0.868 (0.726–1.038)		0.120		0.909 (0.805–1.027)		0.124	0.887 (0.740–1.064)		0.198		0.880 (0.775–1.000)		0.049
Grade														
I and II	Reference				Reference			Reference				Reference		
III and IV	1.613 (1.434–1.813)		< 0.001		1.710 (1.589–1.840)		< 0.001	1.623 (1.439–1.831)		< 0.001		1.742 (1.616–1.878)		< 0.001
Primary site														
Proximal colon	Reference				Reference			Reference				Reference		
Distal colon	0.705 (0.605–0.822)		< 0.001		0.653 (0.602–0.708)		< 0.001	0.694 (0.594–0.812)		< 0.001		0.636 (0.585–0.692)		< 0.001
Rectum	0.585 (0.518–0.662)		< 0.001		0.591 (0.538–0.650)		< 0.001	0.569 (0.502–0.645)		< 0.001		0.577 (0.523–0.636)		< 0.001
Other	1.410 (0.941–2.114)		0.096		1.274 (1.023–1.586)		0.030	1.408 (0.932–2.128)		0.104		1.303 (1.044–1.627)		0.019
Histology type														
AC	Reference				Reference			Reference				Reference		
MAC	1.339 (1.018–1.762)		0.037		1.308 (1.167–1.465)		< 0.001	1.308 (0.983–1.740)		0.065		1.342 (1.195–1.507)		< 0.001
SRCC	1.768 (1.275–2.452)		0.001		2.124 (1.666–2.707)		< 0.001	1.820 (1.307–2.536)		< 0.001		2.160 (1.685–2.769)		< 0.001
CEA														
Normal	Reference				Reference			Reference				Reference		
Abnormal	1.345 (1.149–1.575)		< 0.001		1.563 (1.429–1.708)		< 0.001	1.356 (1.152–1.595)		< 0.001		1.547 (1.412–1.695)		< 0.001
Radiotherapy														
No	Reference				Reference			Reference				Reference		
Yes	0.814 (0.722–0.918)		0.001		0.590 (0.520–0.670)		< 0.001	0.785 (0.693–0.889)		< 0.001		0.596 (0.524–0.678)		< 0.001
Chemotherapy														
No	Reference				Reference			Reference				Reference		
Yes	0.317 (0.281–0.357)		< 0.001		0.359 (0.334–0.387)		< 0.001	0.319 (0.282–0.360)		< 0.001		0.368 (0.341–0.397)		< 0.001
T stage														
T1 and T2	Reference				Reference			Reference				Reference		
T3 and T4	0.949 (0.856–1.052)		0.317		1.438 (1.204–1.719)		< 0.001	0.928 (0.835–1.031)		0.165		1.466 (1.219–1.764)		< 0.001
N stage														
N0	Reference				Reference			Reference				Reference		
N1	0.899 (0.809–1.000)		0.050		1.192 (1.073–1.325)		0.001	0.896 (0.804–1.000)		0.049		1.233 (1.104–1.375)		< 0.001
N2	0.749 (0.610–0.919)		0.006		1.615 (1.461–1.786)		< 0.001	0.735 (0.595–0.908)		0.004		1.698 (1.529–1.885)		< 0.001
Bone metastasis														
No	Reference				Reference			Reference				Reference		
Yes	1.736 (1.453–2.073)		< 0.001		1.745 (1.460–2.085)		< 0.001	1.698 (1.412–2.042)		< 0.001		1.762 (1.468–2.115)		< 0.001
Liver metastasis														
No	Reference				Reference			Reference				Reference		
Yes	1.029 (0.910–1.162)		0.650		1.164 (1.076–1.260)		< 0.001	1.075 (0.947–1.220)		0.266		1.181 (1.089–1.281)		< 0.001
Lung metastasis														
No	Reference				Reference			Reference				Reference		
Yes	1.073 (0.965–1.193)		0.196		1.211 (1.111–1.319)		< 0.001	1.086 (0.974–1.211)		0.139		1.206 (1.104–1.317)		< 0.001
Brain metastasis														
No	Reference				Reference			Reference				Reference		
Yes	2.027 (1.393–2.950)		< 0.001		2.021 (1.352–3.020)		0.001	2.072 (1.414–3.037)		< 0.001		1.967 (1.293–2.993)		0.002

*CI* confidence interval, *AC* adenocarcinoma, *MAC* mucinous adenocarcinoma, *SRCC* signet ring cell carcinoma, *PTR* primary tumor resection

**Table 5:** Multivariable Cox regression analyses for metastasis colorectal cancer patients in PTR and non-PTR groups

Variables	Overall survival				Cancer-specific survival			
	Non-PTR		PTR		Non-PTR		PTR	
	HR (95% CI)	*p* value	HR (95% CI)	*p* value	HR (95% CI)	*p* value	HR (95% CI)	*p* value
Age								
< 60	Reference		Reference		Reference		Reference	
≥ 60	1.363 (1.224–1.517)	< 0.001	1.265 (1.175–1.361)	< 0.001	1.342 (1.203–1.498)	< 0.001	1.240 (1.150–1.337)	< 0.001
Gender								
Male	–		Reference		–		Reference	
Female	–		1.082 (1.009–1.160)	0.026	–		1.081 (1.006–1.161)	0.033
Race								
Black	Reference		–		Reference		Reference	
White	1.192 (1.037–1.371)	0.014	–		1.177 (1.019–1.359)	0.027	1.049 (0.951–1.157)	0.338
Other	0.837 (0.699–1.004)	0.055	–		0.860 (0.715–1.033)	0.107	0.930 (0.819–1.057)	0.270
Grade								
I and II	Reference		Reference		Reference		Reference	
III and IV	1.632 (1.439–1.851)	< 0.001	1.601 (1.483–1.729)	< 0.001	1.638 (1.440–1.864)	< 0.001	1.620 (1.497–1.752)	< 0.001
Primary site								
Proximal colon	Reference		Reference		Reference		Reference	
Distal colon	0.785 (0.670–0.919)	0.003	0.766 (0.704–0.833)	< 0.001	0.769 (0.654–0.904)	0.001	0.748 (0.686–0.816)	< 0.001
Rectum	0.628 (0.547–0.720)	< 0.001	0.830 (0.746–0.922)	0.001	0.614 (0.534–0.707)	< 0.001	0.809 (0.725–0.903)	< 0.001
Other	1.156 (0.769–1.739)	0.486	1.099 (0.882–1.370)	0.399	1.147 (0.756–1.740)	0.519	1.131 (0.904–1.413)	0.281
Histology type								
AC	Reference		Reference		Reference		Reference	
MAC	1.091 (0.825–1.443)	0.539	1.235 (1.100–1.387)	< 0.001	1.055 (0.789–1.411)	0.717	1.263 (1.122–1.422)	< 0.001
SRCC	1.133 (0.805–1.596)	0.474	1.720 (1.335–2.215)	< 0.001	1.163 (0.821–1.647)	0.395	1.748 (1.348–2.266)	< 0.001
CEA								
Normal	Reference		Reference		Reference		Reference	
Abnormal	1.357 (1.156–1.592)	< 0.001	1.554 (1.419–1.703)	< 0.001	1.365 (1.158–1.610)	<0.001	1.533 (1.396–1.683)	< 0.001
Radiotherapy								
No	Reference		Reference		Reference		Reference	
Yes	0.983 (0.856–1.129)	0.807	0.819 (0.710–0.945)	0.006	0.952 (0.825–1.099)	0.501	0.838 (0.723–0.970)	0.018
Chemotherapy								
No	Reference		Reference		Reference		Reference	
Yes	0.304 (0.268–0.344)	< 0.001	0.382 (0.354–0.412)	< 0.001	0.305 (0.268–0.347)	< 0.001	0.389 (0.359–0.421)	< 0.001
T stage								
T1 and T2	–		Reference		–		Reference	
T3 and T4	–		1.152 (0.960–1.382)	0.128	–		1.149 (0.951–1.388)	0.150
N stage								
N0	Reference		Reference		Reference		Reference	
N1	1.045 (0.935–1.167)	0.439	1.170 (1.051–1.301)	0.004	1.044 (0.932–1.170)	0.455	1.203 (1.076–1.345)	0.001
N2	0.934 (0.759–1.150)	0.521	1.482 (1.336–1.644)	< 0.001	0.922 (0.743–1.143)	0.457	1.551 (1.393–1.728)	< 0.001
Bone metastasis								
No	Reference		Reference		Reference		Reference	
Yes	1.751 (1.454–2.108)	< 0.001	1.653 (1.379–1.980)	< 0.001	1.722 (1.421–2.088)	< 0.001	1.668 (1.386–2.008)	< 0.001
Liver metastasis								
No	–		Reference		–		Reference	
Yes	–		1.380 (1.269–1.501)	< 0.001	–		1.408 (1.290–1.535)	< 0.001
Lung metastasis								
No	–		Reference		–		Reference	
Yes	–		1.366 (1.251–1.492)	< 0.001	–		1.375 (1.255–1.506)	< 0.001
Brain metastasis								
No	Reference		Reference		Reference		Reference	
Yes	1.420 (0.959–2.101)	0.080	1.483 (0.983–2.236)	0.060	1.483 (0.995–2.212)	0.053	1.462 (0.952–2.246)	0.083

*CI* confidence interval, *AC* adenocarcinoma, *MAC* mucinous adenocarcinoma, *SRCC* signet ring cell carcinoma, *PTR* primary tumor resection

### Individualized construction of nomograms

**Fig. 3:**
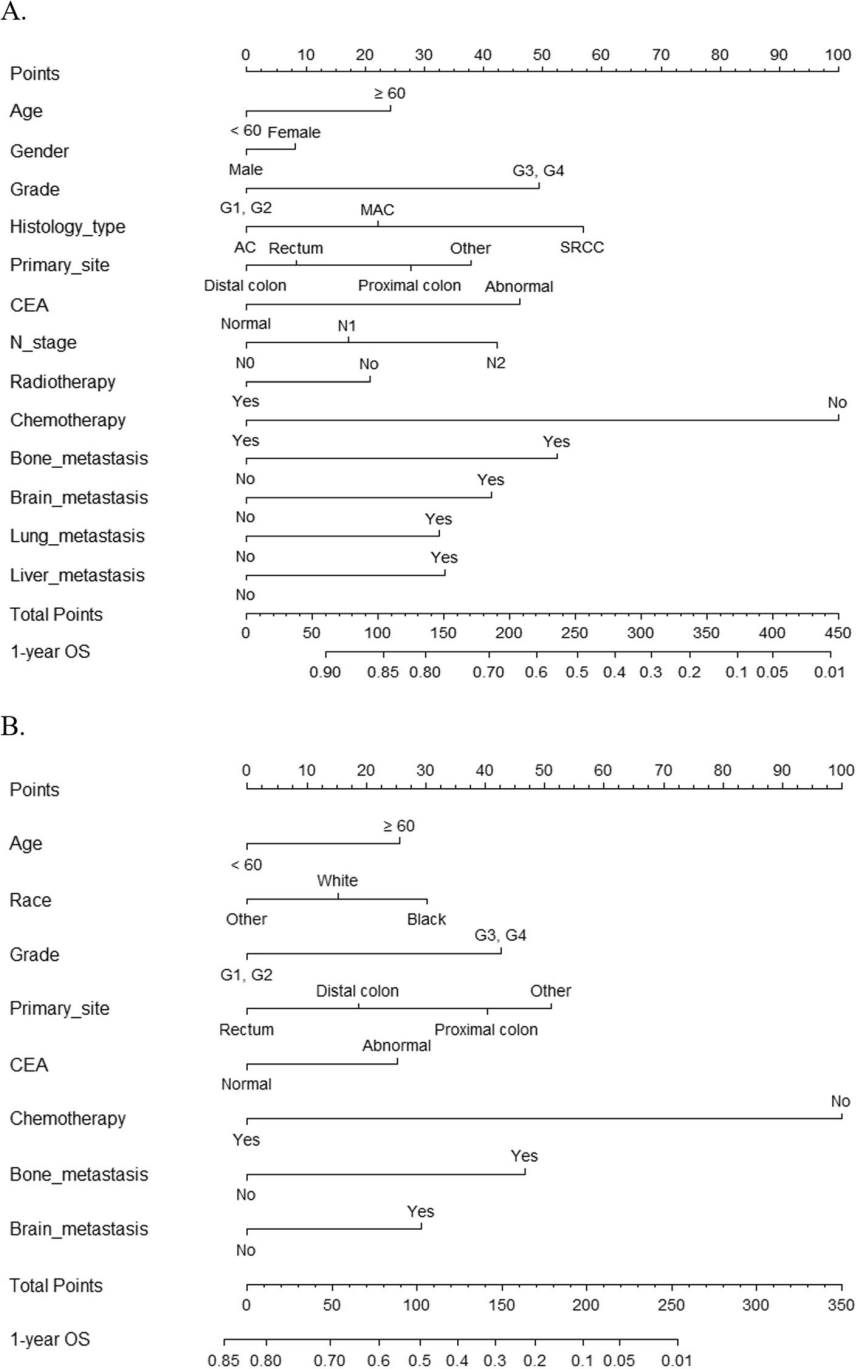
Nomograms for predicting 1-year overall survival (OS) in patients with unresectable metastatic colorectal cancer. **a** OS for patients with primary tumor resection. **b** OS for patients without primary tumor resection

Based on aforementioned independent prognostic factors in multivariable Cox analyses and the minimum AIC values, two nomograms were constructed to predict the 1-year OS in PTR group and non-PTR group, respectively (Fig. 3). The prediction websites of the nomograms predicting the 1-year OS in PTR group and non-PTR group are https://crcnomograma.shinyapps.io/NomogramIVCRCPTR/ and https://crcnomogramb.shinyapps.io/NomogramIVCRCnon-PTR/. By adding up the scores of the factors included in the nomograms, each patient could get two total scores from these separate nomograms that could evaluate the probabilities of 1-year OS of patients when receiving PTR or not, respectively. In additionally, two nomograms predicting the 1-year CSS of patients when receiving PTR and not receiving PTR are shown in Additional file Figure 1. The prediction websites of the nomograms predicting the 1-year CSS in PTR group and non-PTR group are https://crcnomograma.shinyapps.io/NomogramIVCRCPTRCSS/ and https://crcnomogramb.shinyapps.io/NomogramIVCRCnon-PTRCSS/. By comparing the probabilities of survival predicted by those nomograms, we could further predict whether patients might benefit from PTR.

### Efficacy of the predictive models

**Fig. 4:**
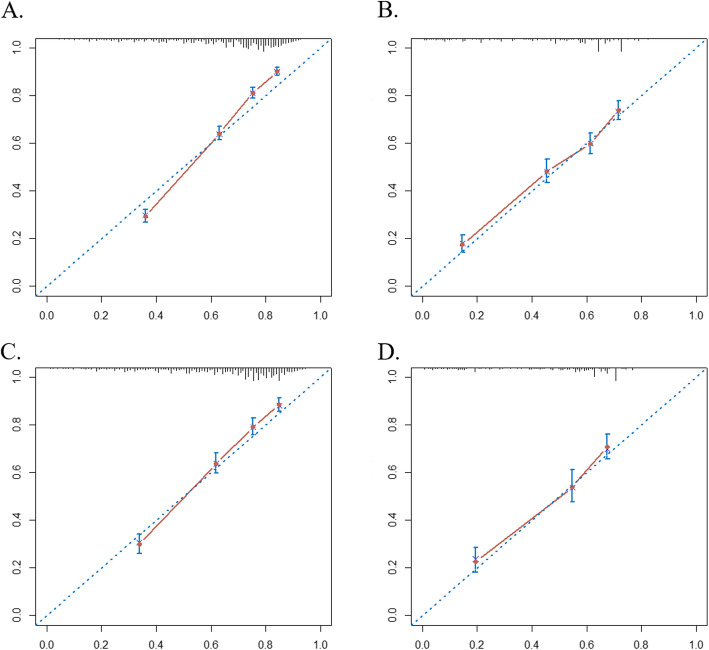
Calibration curves of the nomograms for predicting 1-year overall survival **a** for patients with primary tumor resection (PTR) in training cohort, **b** for patients without PTR in training cohort, **c** for patients with PTR in validation cohort, and **d** for patients without PTR in validation cohort

**Fig. 5:**
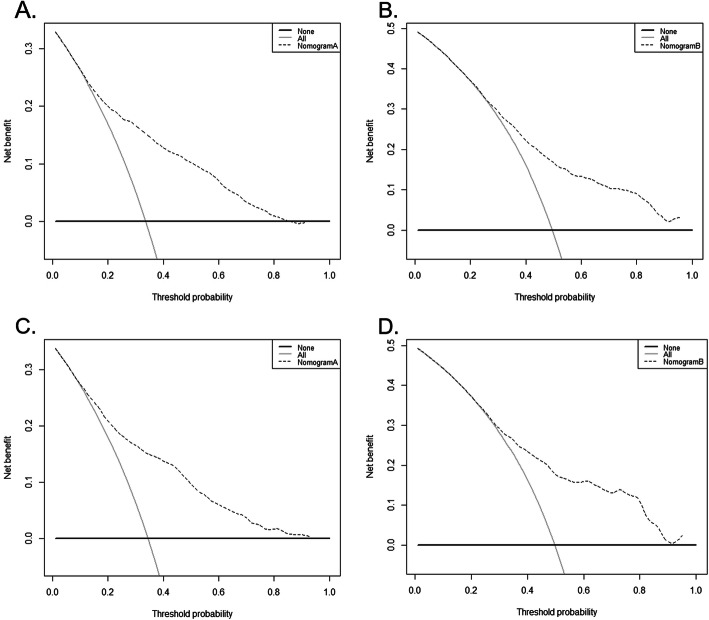
Decision curve analyses for the nomograms in regard to 1-year overall survival **a** for patients with primary tumor resection (PTR) in training cohort, **b** for patients without PTR in training cohort, **c** patients with PTR in validation cohort, and **d** for patients without PTR in validation cohort

The C-indices of the nomograms to separately predict the OS and CSS in PTR group were 0.719 (95% CI 0.710–0.729) and 0.720 (95% CI 0.710–0.729) in the training cohort and 0.725 (95% CI 0.711–0.739) and 0.722 (95% CI 0.708–0.736) in the validation cohort, respectively. Furthermore, the C-indices that corresponded to the nomograms in non-PTR group were 0.701 (95% CI 0.687–0.717), 0.700(95% CI 0.684–0.715), 0.706 (95% CI 0.684–0.729), and 0.705 (95% CI 0.682–0.727). Calibration curve for the two nomograms predicting the OS showed pronounced agreement between prediction and observation in both training and validation cohorts (Fig. 4). Additionally, the DCAs exhibited the ideal net benefits in all patients for predicting OS, an indication that these nomograms had good clinical value (Fig. 5). The calibration curves (Additional file Figure 2) and the DCAs (Additional file Figure 3) for the two nomograms predicting the CSS suggested the models had good performance.

## Discussion

In this study, we identified independent risk factors associated with the prognosis of unresectable mCRC patients in PTR group and non-PTR group. In addition, we established and validated several customized nomograms to predict survival of patients when receiving PTR or not, respectively. These nomograms could help clinicians predict whether patients could obtain survival benefit from PTR by comparing the predictable survival of unresectable mCRC patients when receiving PTR or not before surgery, thereby making reliable clinical decisions and optimize disease management.

Generally, PTR is recommended for patients with fatal complications. A previous meta-analysis including 1062 patients reported 87% of unresectable stage IV patients with tumor-related complication required surgical intervention, thereby underlining the importance of PTR to such patients [23]. However, emergent surgery has been proved to be associated with the great risk and poor oncological outcome [24–26]. Besides, a symptomatic obstruction that requires surgery occurs in approximately 20% of patients before death [11, 18]. PTR prior to the onset of symptoms may effectively reduce the tumor volume to prevent future morbidity and mortality associated with these tumor-related complications [6, 7]. PTR can also decrease protumorigenic mediators in circulation, which may affect tumor growth and angiogenesis [27]. A recent meta-analysis of unresectable mCRC patients from 17 retrospective studies by Ha et al. revealed significantly improved survival associated with PTR [28]. Despite the benefits of PTR, it may delay the time in the administration of chemotherapy and increases the potential risk of surgery-related complications, which may subsequently increase death risk [13–16].

The impact of PTR on the prognosis of unresectable mCRC patients remains controversial [5, 8–12, 17–22]. Our study showed that not all patients are guaranteed of the survival benefits. Considering that all existing studies assessing the impact of PTR on the prognosis, including our study, are retrospective with selection biases and heterogeneity, the impact of surgery on the prognosis was still unable to be accurately assessed. Some authors have attributed the different survival benefits after PTR to disparities in characteristics of the patients [29]. Therefore, accurate and feasible prognostic models based on the characteristics of the patients are needed as a reference to help clinicians identify candidates who are likely to get survival benefit from PTR. However, it should be noted that these models cannot replace the outcome of prospective randomized clinical trials.

To better know the prognosis of the patients with and without PTR, we identified the relationship between clinicopathologic characteristics and survival of unresectable mCRC patients in PTR and non-PTR groups, respectively. There were some differences in prognostic factors between PTR group and non-PTR group. Eight variables (age, race, tumor grade, primary site, CEA, chemotherapy, brain metastasis, and bone metastasis) and seven variables (age, race, tumor grade, primary site, CEA, chemotherapy, and bone metastasis) were separately identified to be associated with the OS and CCS of unresectable mCRC patients without surgery respectively, while 13 variables (age, gender, tumor grade, tumor site, N stage, CEA, chemotherapy, radiotherapy, histological type, brain metastasis, liver metastasis, lung metastasis, and bone metastasis) were identified to be related to the survival in the PTR group.

Intriguingly, radiotherapy was only a protective factor in the PTR group. Preoperative radiotherapy can improve the primary tumor to make lesions more resectable [30–32]. Radiotherapy combined with PTR is feasible and can improve the survival of mCRC. However, we found radiotherapy did not improve the survival of patients without PTR. The mechanism behind this finding remains unclear and requires further examination. Therefore, cautious interpretation of this result needs to be warranted. Chemotherapy, as an important and effective method, is preferentially recommended for mCRC patients by various guidelines [6, 7], which were proved to be the protective factor in both PTR and non-PTR groups in our study. Although PTR may delay chemotherapy, which could decrease the latter’s efficacy [13–16], PTR could also reduce tumor loading to improve response rates to chemotherapy [27]. Therefore, decision-making should balance the surgery-related benefits and risk.

Based on the results of multivariable Cox analyses and the minimum AIC values, we constructed several individualized nomograms in the training cohort to predict the survival of unresectable mCRC patients in the PTR and non-PTR groups, respectively. From the perspective of C-index and the calibration plots in the training and validation cohorts, the nomograms exhibited reliable discrimination and calibration ability. Moreover, these nomograms showed good clinical value, as revealed by DCA. Individualized risk predictive models with great predictive performance could assist clinicians and patients in deciding whether or not PTR would be the best choice with reference to predictable survival. Therefore, clinicians may select candidates likely to get survival benefit from PTR before the surgery.

Several prognostic prediction models for unresectable mCRC patients have been previously reported [33–35]. Li et al. developed a prognostic scoring system known as AAAP including age, ALP, ascites, and PLR based on 110 patients [33]. Cao et al. developed another scoring system including four variables (CEA, NLR, LDH, and CA19-9) based on 138 patients [34]. Variables, such as NLR, LDH, and PLR, might be easily influenced by infection. In addition, Dorajoo and the colleagues developed a scoring model including some clinicopathological characteristics to predict the survival of patients with PTR based on the 379 patients [35]. However, all these previous studies were conducted from respective single-centers and had small sample sizes [33–35]. Besides, they lacked a comparison group to predict the survival of patients without PTR. We constructed nomograms that could independently predict the survival of patients in PTR and non-PTR groups. By comparing the predictable survival of patients when receiving PTR or not, clinicians may predict the patients whether could get survival benefit from the PTR. In addition, the large sample size utilized to make our nomograms more practical, reliable, and accurate compared with the previous models. To the best of our knowledge, this is the first study using nomograms to predict the survival of unresectable mCRC patients. A nomogram is a practical tool that can present a wide range of threshold probabilities and output the patient’s prognosis visually.

Despite the advantages of our study, several potential limitations should also be considered. First, the SEER database lacked detailed information, such as the physical conditions, molecular-targeted therapy, the sequence of chemotherapy, and peritoneal metastasis. Secondly, given that this was a retrospective study, selection bias might be inherent. Besides, we speculate that retrospective studies might not fully assess the survival benefits of PTR to unresectable mCRC. Therefore, future prospective randomized controlled trials should focus on providing more valuable evidence on this phenomenon. Thirdly, our nomograms were only validated in an internal cohort. Therefore, external validation of these nomograms and the prospective evaluation of their clinical translation are required.

## Conclusions

Our findings suggested most patients with unresectable mCRC had potential survival benefits to PTR. However, given the retrospective nature of our study, we were unable to fully assess the impact of PTR on the survival of unresectable mCRC patients. Therefore, we further developed and validated several individualized nomograms that could separately predict 1-year survival of unresectable mCRC patients with or without PTR, respectively. These nomograms could assist clinicians in making appropriate treatment decisions to effectively manage the disease by comparing the predictable survival of patients with and without PTR before surgery.

## Supplementary information

**Additional file 1: Fig S1.** Nomograms for predicting 1-year cancer-specific survival (CSS) in patients with unresectable metastatic colorectal cancer (a) CSS for patients with primary tumor resection (b) CSS for patients without primary tumor resection.

**Additional file 2: Fig S2.** Calibration curves of the nomograms for predicting 1-year cancer-specific survival (CSS) (a) for patients with primary tumor resection (PTR) in training cohort, (b) for patients without PTR in training cohort, (c) for patients with PTR in validation cohort, and (d) for patients without PTR in validation cohort.

**Additional file 3: Fig S3.** Decision curve analyses for the nomograms in regard to 1-year cancer-specific survival (CSS) (a) for patients with primary tumor resection (PTR) in training cohort, (b) for patients without PTR in training cohort, (c) patients with PTR in validation cohort and (d) for patients without PTR in validation cohort.

## Data Availability

The data for constructing model were obtained from the SEER database.
