# Supplementary figures and images for: Individualized prediction of survival benefit from primary tumor resection for patients with unresectable metastatic colorectal cancer
Source: World J Surg Oncol. 2020 Aug 3;18:193. doi: 10.1186/s12957-020-01972-y (PMC7401291; doi:10.1186/s12957-020-01972-y)

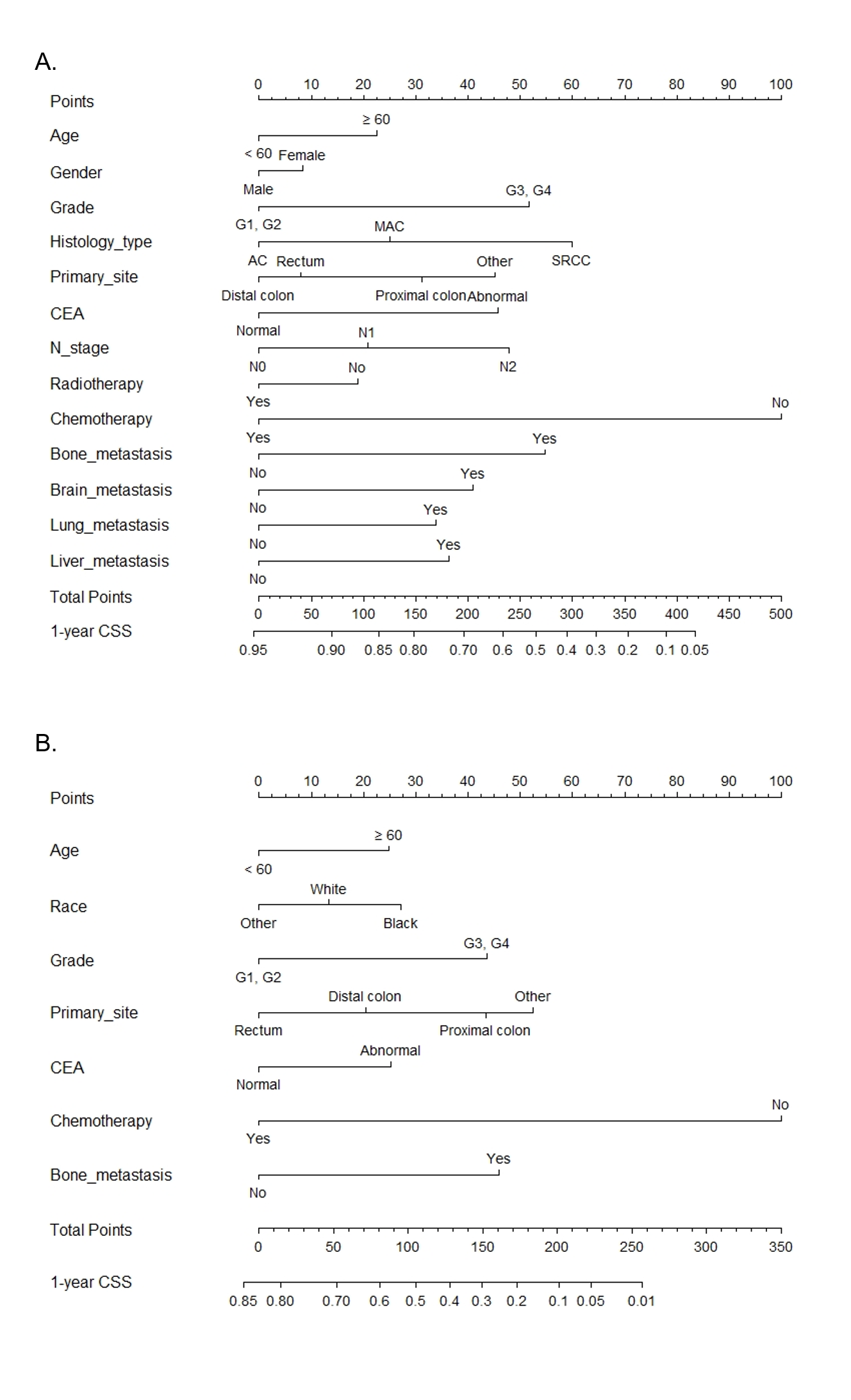

Supplement: Supplementary file 1 — Additional file 1: Fig S1. Nomograms for predicting 1-year cancer-specific survival (CSS) in patients with unresectable metastatic colorectal cancer (a) CSS for patients with primary tumor resection (b) CSS for patients without primary tumor resection. [file 12957_2020_1972_MOESM1_ESM.tif]

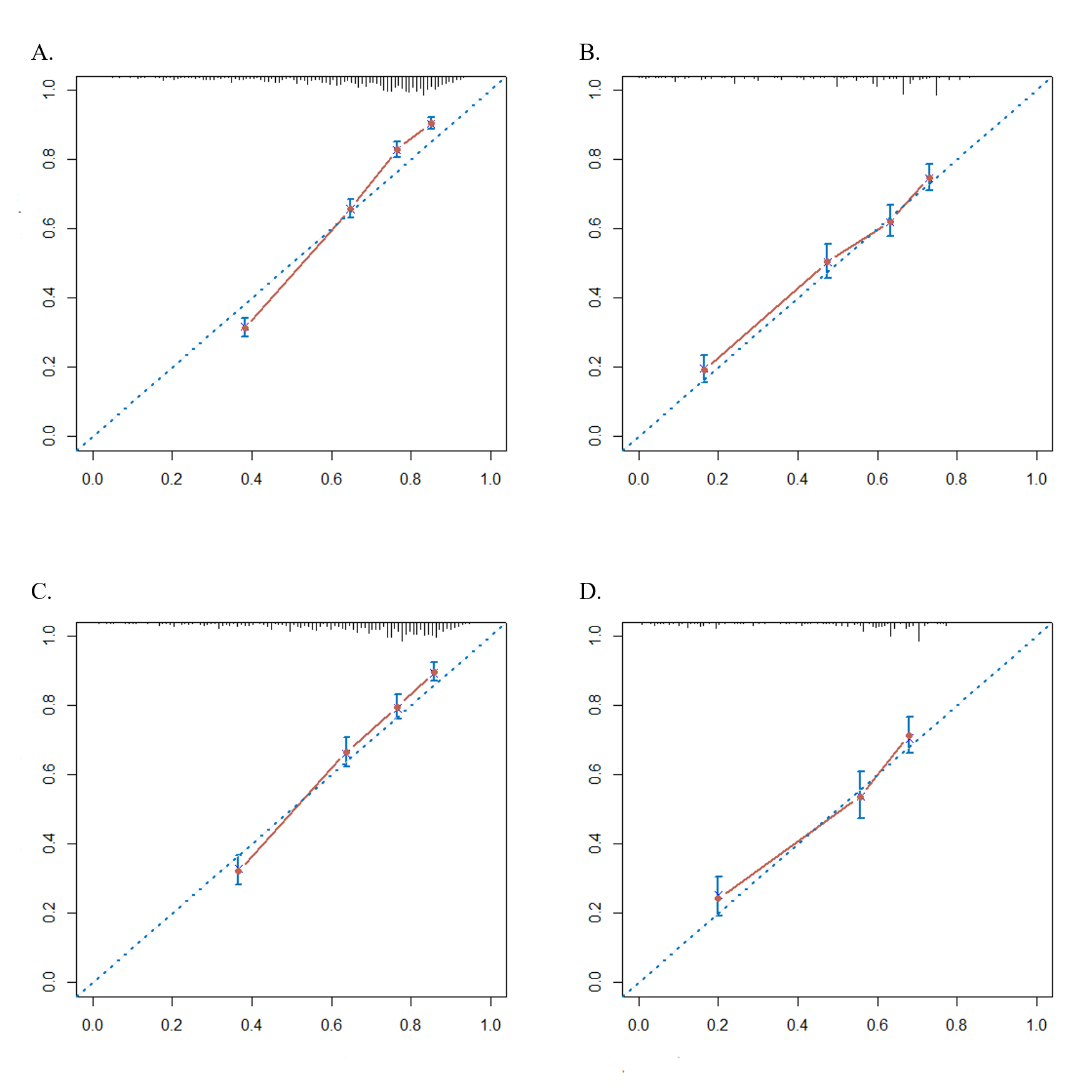

Supplement: Supplementary file 2 — Additional file 2: Fig S2. Calibration curves of the nomograms for predicting 1-year cancer-specific survival (CSS) (a) for patients with primary tumor resection (PTR) in training cohort, (b) for patients without PTR in training cohort, (c) for patients with PTR in validation cohort, and (d) for patients without PTR in validation cohort. [file 12957_2020_1972_MOESM2_ESM.tif]

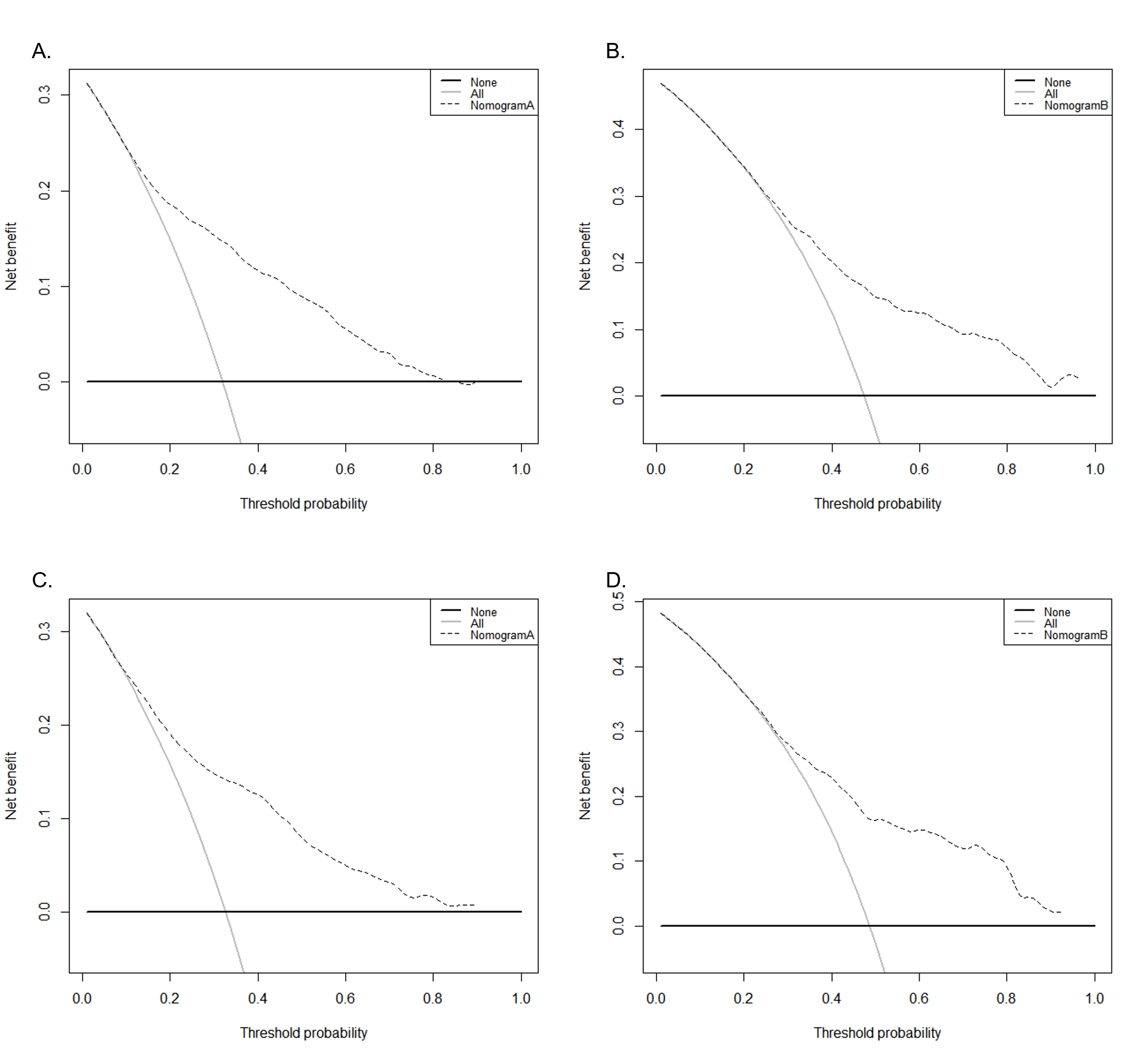

Supplement: Supplementary file 3 — Additional file 3: Fig S3. Decision curve analyses for the nomograms in regard to 1-year cancer-specific survival (CSS) (a) for patients with primary tumor resection (PTR) in training cohort, (b) for patients without PTR in training cohort, (c) patients with PTR in validation cohort and (d) for patients without PTR in validation cohort. [file 12957_2020_1972_MOESM3_ESM.tif]
